# Re‐considering and designing microbiomes for future waste biorefinery

**DOI:** 10.1111/1751-7915.14395

**Published:** 2024-01-11

**Authors:** Menghan Wu, Hui Wang

**Affiliations:** ^1^ State Key Joint Laboratory of Environmental Simulation and Pollution Control, School of Environment Tsinghua University Beijing China

## Abstract

It is an increasingly promising research direction using microbiomes to produce various chemicals in order to support people's growing need for sustainability. Currently, bottom‐up constructed defined microbiomes and top‐down constructed undefined microbiomes play an essential role in the fields of synthetic biology and environmental engineering, respectively. However, if we are goal‐oriented and want to align scientific principles with technology and engineering in future waste biorefinery, we need to reconsider and design microbiomes interdisciplinarily. In this editorial, we briefly review the latest applications of two approaches to microbiome design (bottom‐up and top‐down) and the dilemmas faced in using complex waste. Consequently, we introduce the concept of ‘sustainable synthetic microbiomics’ to apply combined bottom‐up and top‐down constructed microbiomes to provide products for human needs from low‐value waste. Furthermore, we outline the relatively comprehensive research contents and expected prospects based on the pressing problems. Finally, burning questions on key research contents are proposed for specific cases, hoping to provide valuable views for future microbiome biorefinery.

## CURRENT STATUS OF MICROBIOMES IN WASTE BIOREFINERY

Synthetic biology and metabolic engineering utilising advanced molecular biology techniques, genomic information and computer technology have achieved the production of 532 compounds (Jang et al., 2023), natural or unnatural, covering the fields of food, medicine, environment, daily necessities, cosmetics and construction. Synthetic biology and metabolic engineering, as the novel intersection of biology and engineering, have been supported by various bioeconomic policies worldwide and extensively promote biomanufacturing. Biomanufacturing usually consists of three significant links: substrate selection, microorganism establishment and engineering implementation (including process route, operation parameter control, end‐product extraction and waste treatment). Especially, the establishment of microorganisms becomes the focus of research and engineering because the rational design of specific microbes in synthetic biology remoulds the traditional fermentation process. However, when facing complex waste substrates rather than crop‐based substrates, employing microbiomes instead of a single engineered strain to produce desired compounds is considered to have superior application potential. One of the most important reasons is that using microbiomes can exert the strengths of multiple populations to separate metabolic steps rationally through the division of labour (Tsoi et al., 2018), thus avoiding the complexity of genetic manipulation, metabolic stress and genetic loss imposed on the strain. Therefore, research on microbiome engineering and waste biorefinery has been on the rise in recent years (Goers et al., 2014).

There are two ways to design and construct microbiomes: bottom‐up and top‐down (Lawson et al., 2019). Based on the existing genetic and functional knowledge of pure strains, bottom‐up methods combine different defined microbial populations that undertake interrelated metabolic functions rationally according to a pre‐determined route. The design principle of the bottom‐up microbiome is mainly to establish a cooperative relationships in the most minimalist degree, which includes the upstream and downstream metabolic link relationship and the auxiliary relationship. As for biofuel or chemical production from lignocellulosic biomass, two synthetic modules, including hydrolytic fungi and fermenting bacteria based on metabolic upstream and downstream relationships, are usually adopted to refine the waste (Jiménez et al., 2018; Minty et al., 2013). In an energy power generation system, *Bacillus subtilis* is designed to produce riboflavin, providing for *Shewanella oneidensis*, which serves as the exoelectrogen to generate electricity, in which the riboflavin only auxiliarily strengthens the function of *Shewanella oneidensis* and does not undertake a mainstream role in the metabolic route. To broaden the number of synthetic modules and the application scope of defined microbiomes, Shahab et al. (2020) created spatially heterogeneous ecological niches of aerobe (hydrolysis), amphicicrobe (lactic acid production) and anaerobion (volatile fatty acid fermentation) by adjusting the oxygen diffusion gradient within a heterogeneous reactor. Therefore, they could play a role in their own space and convert lignin or beech into volatile fatty acid (VFA) by cooperating with three microbial modules. However, bottom‐up microbiome construction for chemical production still requires sterility of the fermentation environment, and no more than four microbial strains are currently designed and established in one biosynthesis reactor due to the lack of complex ecological mechanisms and feasible technology.

The use of self‐organised undefined microbiomes by the top‐down method is preferred if the biorefinery process is carried out in an open, low‐energy process without stringent sterilisation. The reference scenario for undefined microbiomes is the wastewater treatment plant (WWTP), which has nearly 1000 genera and 1500 species abundant globally (Dueholm et al., 2022). Nonetheless, the production of diverse chemicals (e.g. lactic acid, ethanol, methanol, short‐chain, medium‐chain and long‐chain fatty acid, hydrogen, plastics) has been successfully realised using undefined microbiomes by top‐down methods. Top‐down constructed microbiomes have several characteristics compared to bottom‐up constructed microbiomes: high functional redundancy, self‐organisation dependent on environmental pressure, complex species interactions and frequent migration of microbes with an external open environment. Due to these characteristics, it is possible to refine more complex wastes (e.g., food waste, industrial wastewater, waste activated sludge) and to improve the economy of fermentation by eliminating complex sterilisation operations. However, most products synthesised by undefined microbiomes have been limited to primary products and seldom involve intermediate metabolites (Scarborough et al., 2022). In addition, it is also difficult to precisely control the desired production if there are various end‐products, for example, a specific species of fatty acids. These difficulties are partly because refining processes using undefined microbiomes is passive in performing specific tasks under adjusted environmental conditions and needs more active and rational pre‐design, which is a necessary step in bottom‐up constructed defined microbiomes.

The above discussed bottom‐up constructed defined and top‐down constructed undefined microbiomes have similarities and differences, as well as their own advantages and disadvantages when applied to biomanufacturing. It is foreseeable that both approaches will slowly converge, making up for their respective shortcomings while bringing their strengths to a qualitative rise and breakthrough. In an exemplary study in the medical field, Cheng et al. (2022) created a well‐defined and high‐complexity microbiome containing 119 strains. This microbiome achieved successful colonisation of the mouse intestine and effectively resisted the invasion of enterohemorrhagic *Escherichia coli* ATCC 43894 (EHEC). This is an innovative attempt to combine top‐down data analysis and bottom‐up colony construction. Also, it indicates that as the complexity of the defined microbiome increases, the robustness of defined microbiomes may be closer to that of undefined microbiomes. To sum up, a more precise roadmap is needed in relevant fields to reconsider and design microbiomes for the future waste biorefinery.

**FIGURE 1:**
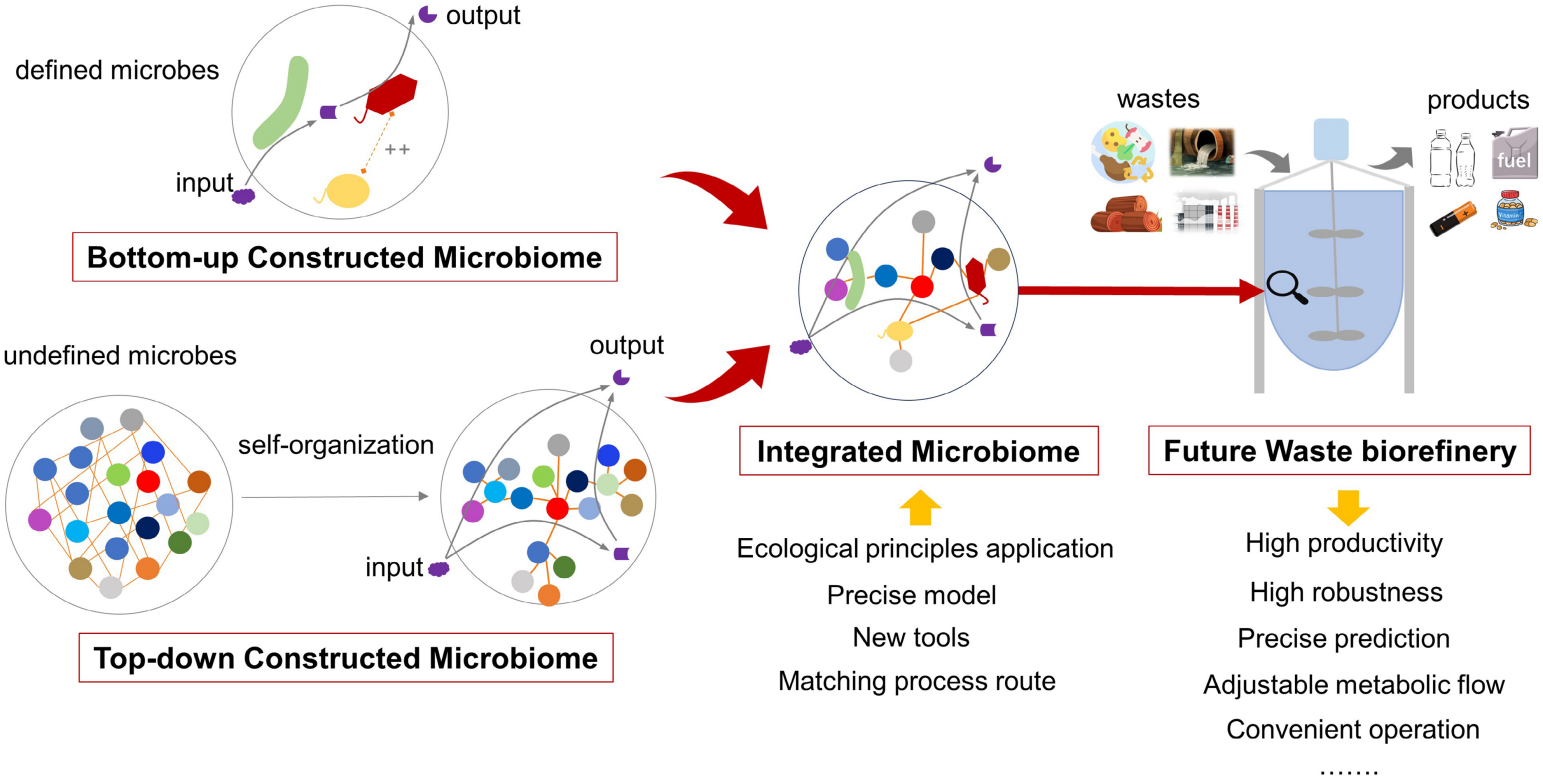
Development trend of microbiomes in future waste biorefinery.

Considering the current frontiers of microbiome engineering in waste biorefinery, we propose a new integrative concept ‘sustainable synthetic microbiomics’ (Figure 1). This concept involves applying combined bottom‐up and top‐down constructed microbiomes to provide products for human needs from low‐value waste. The aim is to achieve high productivity, precise prediction and control, convenient operation and other goals that can improve economic, environmental and social sustainability. Integrating perspectives of ‘science‐technology‐engineering’, we believe that the future research in the sustainable synthetic microbiomics should include, but is not limited to, (1) the application of microbial ecology in microbiome anabolisms, including the selection and construction of core functional microbial population and the robustness of microbial functions; (2) precise model control and prediction in metabolic flow and products varieties; (3) new tools for monitoring, analysing and integrating microbial data and (4) the establishment of matching process modes and routes.

## BURNING IDEAS FOR THE FUTURE RESEARCH CONTENTS

The waste biorefinery application of microbiomes is quite different from other microbiomes' applications (such as degrading toxic and harmful substances, improving soil health and assisting in treating human diseases). Biorefinery has a long‐chain production process that aims to get the required products, which requires systematic life cycle thinking and design. Importantly, anabolism is primarily a process of entropy reduction, imposing a higher request for microbiomes. Therefore, we believe that the following burning questions need to be paid attention to in future applications.

Balancing functional robustness and productivity is a unique problem in waste biorefinery using microbiomes. On the one hand, the complex and stable interspecific relationships make the system function robust and can withstand the impact of an open environment. On the other hand, a simple synthetic community or pure culture allows high‐yielding bacteria to function, resulting in high productivity. In polyhydroxyalkanoate (PHA) production using microbiomes, only a few populations with high PHA‐accumulating capacity are essential. Other populations are not introduced intentionally, but their existence can be inferred as a result of maintaining the functional stability of the system and interacting with functional populations (Diaz‐Colunga et al., 2022). Researchers apply varied ‘feast‐famine’ regimes to minimise the number of fluctuation populations with low or no PHA‐accumulation capacity to improve PHA productivity further. From this point of view, we need to innovatively apply ecological theories to create an optimised and simplified community that can ensure the dominance of high PHA‐yielding strains (functional redundancy) while maintaining population structure stability in the face of the migration and migration of flanking species.

Biomass concentration is also a key factor determining productivity and process economy in biorefinery. The traditional production of bulk bio‐based chemicals uses high‐biomass density *Escherichia coli* or yeast fermentation to increase volume productivity and reduce production costs. However, there still needs to be more research on high biomass density microbiomes for waste biorefinery. The interactions between microbial communities will probably differ at high and low biomass densities, leading to ecological principles found at low biomass density failing at high density. Therefore, exploring ecological principles and applying novel engineering strategies under high biomass density is necessary for higher production efficiency using microbiomes.

Improving product selectivity is a challenging problem when using top‐down constructed microbiomes. In VFA biorefinery, according to Shahab et al. (2020), bottom‐up constructed microbiomes can realise specific fatty acid production by replacing the specific lactic acid‐fermenting bacteria. However, top‐down constructed microbiomes usually produce mixed products, including acetate, propionate, butyrate and valerate. The reason is that the composition of substrate is complex, and the metabolic potential of microbiomes is versatile, making it hard to control and adjust the specific metabolic flow. The traditional method to regulate the metabolism of undefined microbiomes is to adjust the environmental parameters and then wait for the post‐feedback regulation, making metabolic control blind. Wu et al. (2023) find that the substrate has a crucial effect on VFA selectivity and speculate that lactic acid has a universal metabolic pathway to produce propionic acid through gene information mining. They verified that hypothesis using three different undefined microbiomes and found that lactic acid can promote the relative abundance of propionic acid‐producing specialists in three different undefined microbiomes. Fortunately, benefiting from the emerging available databases, researchers can easily explore the metabolic role of the microbiomes (Ke et al., 2022; Pavlopoulos, 2023). Preliminary rational analysis of microbiomes and the design of biorefinery conditions based on biological data will facilitate our precise system control.

As reviewed by Leggieri et al. (2021), meta‐omics and computer tools can collect and integrate microbial data to better understand synthetic and natural microbiomes and are bases for control and prediction. The development of metagenomics, metatranscriptomics, metaproteomics and metabolomics allows for clarification of the diversity of microorganisms and their functions in different habitats. Moreover, recent advances in third‐generation sequencing technology have dramatically improved the revolution in species and gene structure, which plays a vital role in parsing the gene information of high‐complex microbiomes. However, most of the current studies in biorefinery with top‐down constructed microbiomes are still limited to descriptions based on microbial data rather than general ecological rule discovery and application. This is because the higher the complexity of the system, the more it faces the dual influence caused by the random oscillations of the environment and the intrinsic properties of the complex system (interspecific interaction network). If we can rethink microbial ecosystems across disciplines, with the help of tools such as artificial intelligence, it may be possible to re‐understand and analyse how complex ecosystems work fundamentally. A study published in Science by Hu et al. (2022) is inspired by the theory of statistical physics, demonstrating that species dynamics and phase transitions in complex ecosystems can be predicted with only a tiny number of community‐scale control variables (number of species and average strength of interspecific interactions). Furthermore, by using microbial data at different scales (genes, proteins, species, etc.), novel models can be developed with the assistance of machine learning (Lawson et al., 2021), which has been gradually applied in many fields and substantially enhanced the predictability of microbiomes (Liu et al., 2023; Wang et al., 2023).

With the in‐depth study of the microbiome and cross‐disciplinary application, the design‐build‐test‐learn (DBTL) circle (Lawson et al., 2019) of microbiome engineering will be extensively applied in practice. Currently, it is possible to achieve the automated design of three‐species synthetic microbial systems, and the design and manipulation of more complex microbial communities will be expected to evolve with an integrated understanding of the principles of top‐down and bottom‐up microbiome construction.

## AUTHOR CONTRIBUTIONS

**Menghan Wu:** Conceptualization (equal); writing – original draft (equal); writing – review and editing (equal). **Hui Wang:** Validation (equal); writing – review and editing (equal).

## FUNDING INFORMATION

The Funding Information section should appear even if no funding information is provided by the author. In that case, the text is: No funding information provided.

## CONFLICT OF INTEREST STATEMENT

All authors have no conflict of interest to declare.
